# A Nature-Inspired Search Space Reduction Technique for Spine Identification on Ultrasound Samples of Spina Bifida Cases

**DOI:** 10.1038/s41598-020-66468-x

**Published:** 2020-06-09

**Authors:** Çağlar Cengizler, M. Kerem Ün, Selim Büyükkurt

**Affiliations:** 1 Department of Biomedical Engineering, Faculty of Engineering, Çukurova University, 01330 Adana, Turkey; 2 Department of Obstetrics and Gynaecology, Medical Faculty, Çukurova University, 01330 Adana, Turkey

**Keywords:** Anatomy, Medical research

## Abstract

Spina bifida is a birth defect caused by incomplete closing around the spinal cord. Spina bifida is diagnosed in a number of different ways. One approach involves searching for a deformity in the spinal axis via ultrasound. Although easy to apply, this approach requires a highly trained clinician to locate the abnormality due to the noise and distortion present in prenatal ultrasound images. Accordingly, visual examination of ultrasound images may be error prone and subjective. A computerized support system that would automatically detect the location of the spinal deformity may be helpful to the clinician in the diagnostic process. Such a software system first and foremost would require an algorithm for the identification of the entire (healthy or unhealthy) spine in the ultrasound image. This paper introduces a novel flocking dynamics based approach for reducing the size of the search space in the spine identification problem. Proposed approach accepts bone-like blobs on the ultrasound images as bird flocks and combine them into bone groups by calculating the migration path of each flock. Presented results reveal that the method is able to locate correct bones to be grouped together and reduce search space (i.e. number of bones) up to 68%.

## Introduction

Spina bifida (SB) is a developmental fetal spine defect. The defect is caused by incomplete closure of the embryonic neural column which results to a split deformation on spinal anatomy^1^. (The name of this pathology translates as “split spine”.) It is the most commonly encountered disability after cerebral palsy^2^. SB can be diagnosed with two conventional screening methods before birth. The first method involves screening mother’s blood for alpha-fetoprotein and the second one is based on observing the ultrasound images to identify any existing anatomical abnormality of spine (vertebral ossifications of the fetus becomes detectable on ultrasound images in the early second trimester) or deviations in intracranial anatomy possibly caused by SB^3^. It should be noted that, while three-dimensional ultrasound and magnetic resonance imaging are emerging methods for characterization of the open SB spinal lesion, ultrasound is still the standard diagnostic tool for SB. In the ultrasonic spine examination, the clinician visually looks for signs of an open spine. It is possible to visually observe fetal spine in real time in sagittal, transverse and coronal planes for diagnosis. The diagnosis, as well as the location of the defect, is crucial for parental decision making about possible interventions. It has been shown that if the upper pole of the defect is below the fourth lumbar vertebrae, the estimated prognosis is always better^4^. It has been recently demonstrated that 3D ultrasonography is effective in the diagnosis of the defect and the detection of the upper pole of the lesion^5^. However, 2D or 3D ultrasonography requires a certain level of expertise. (A recent paper underlines the adjacent role of biochemical screening in the diagnosis of fetal SB^6^). Hence, a computerized support system for detecting the location of the spine on the ultrasound images may be helpful in the diagnostic process. For this, the system should be able to automatically identify the spine axis on the sonogram, which in itself would be a challenging task. Since the ultrasound is taken early in the pregnancy, the bony tissues are still in their developmental stage and not very distinguishable on the images due to the distortions and noise on the ultrasound samples^7^. Consequently, conventional morphological image processing methods are likely to be insufficient for effectively locating the bones of the spine axis and a specific algorithm may be more appropriate for the task.

Machine learning based methods are frequently utilized in the literature for coping with automated classification problems on ultrasound images. A support vector machine based algorithm, which is a supervised machine learning approach, is trained to locate the heart in ultrasound images^8^). Similarly, Baumgartner *et al*. have proposed an aided screening system which is based on convolutional neural network^9^. Their novel algorithm provides an estimation for the orientation of certain standard planes in free-hand ultrasound imaging and can automatically produce 13 standard views. A probabilistic boosting-tree based classifier mechanism is utilized for automated segmentation of the object of interest from the background on ultrasound images where the methodology also requires a supervised training stage^10^. In contrast to supervised methods, meta- heuristic methods typically do not require a training stage^11^ and have been employed for ultrasound classification problems. In one such work, unsupervised segmentation of regions from ultrasound samples is performed by employing a meta-heuristic feature selection approach^12^. The work by Tolay *et al*. has been so far the only attempt for spine identification from ultrasound images^7^. (See the Discussion for a comparison with the current work).

While different organs and fetal features (head radius etc.) have been identified on ultrasound images^8–10^, identification of the deformed fetal spine on an ultrasound sample is a problem that is not covered in the literature. To our knowledge, healthy fetal spine axes are detected on relatively clear ultrasound images in one study^7^. The researchers have combined conventional image processing techniques with data clustering approach. Similar deterministic methods have been tried in the preliminary stages of this research, yet failed to produce a reasonable outcome.

The current paper reports the first part of a larger research effort. Here, an innovative nature-inspired algorithm is proposed and evaluated for the reduction of search space on ultrasound samples in the spine identification problem. After segmentation, the ultrasound samples involve an abundant number of binary (black and white) blobs, some of which correspond to neither spine nor even another bone. All possible combinations of blobs (of bone or other) represent the search space, and the combination that includes nothing but all spine bones is the sought optimal solution. In the first stage of the methodology, the search space is reduced in size by combining certain adjacent blobs that are likely to belong to the spine, into a single item. This is accomplished by accepting automatically each extracted bone blob as a bird flock and calculating its “migration path” to other flocks for clustering. The large size of the search space is a problem characteristics that has a direct effect on the classification ability of a meta-heuristic machine learning method^13^. Accordingly, by combining blobs into single items, we effectively decrease the total number of blobs and total number of their possible combinations, thus reducing the size of our search space^14^.

## Methods

In this study, the objective is to present an approach for reducing the complexity of a bone classification problem. Bone regions are extracted from raw ultrasound images by utilizing a series of filters and clustered through a nature-inspired flocking algorithm. To check the effectiveness of this algorithm, spine identification is accomplished through a simple genetic algorithm (GA) utilizing standard genetic operations. In actual problems, the ground truth is not known, yet, we have used the ground truth (set by an expert obstetrician) as fitness measure in GA. By using an idealized fitness criteria based on the ground truth, the performance of the search space reduction algorithm can be evaluated independent from the GA fitness function. An appropriate aim-specific fitness function which is not based on the ground truth is researched in a follow-up paper. Consecutive stages of the study are explained below.

### Preparation of data-set from ultra-sound images

The proposed methodology is tested on sagittal plane images of fourteen different fetal spines, diagnosed with SB with varying degrees of severity by an expert obstetrician. Sample images are acquired with a General Electric 4k ultrasound imaging device (General Electric, Boston, MA) by the same expert at Çukurova University, Faculty of Medicine. (Proposed study is based on non-interventional medical data and the authors declare that this study does not contain any personal information that could lead to the identification of the patients and Informed consent was obtained from all participants. The work described below has been carried out in accordance with the ethical approval of the Çukurova University Faculty of Medicine, Non-Interventional Clinical Research Ethics Committee).

All sample images are processed and filtered during the preprocessing stage. That stage involves basic filtering operations that suppress noise and convert raw images into binary masks where each bone region appears as a blob. It should be noted that fetal spine bones to be located are among these blobs.

The initial step involves blurring the raw gray-scale images with Gaussian filter. The blurred images are then processed with top-hat filter with a disk-shaped structuring element. Next, resulting images are thresholded in order to generate a binary image.

**Figure 1:**
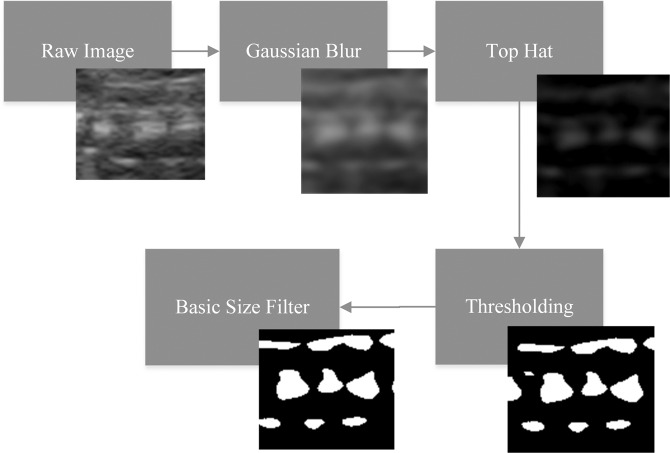
Stages of image preprocessing.

Applied operations remove high frequency noise from the samples and allow us to deal with only bone-like structures on the image^7^. Finally, a blob size filter is applied to the image for removing small objects (Fig. 1).

**Figure 2:**
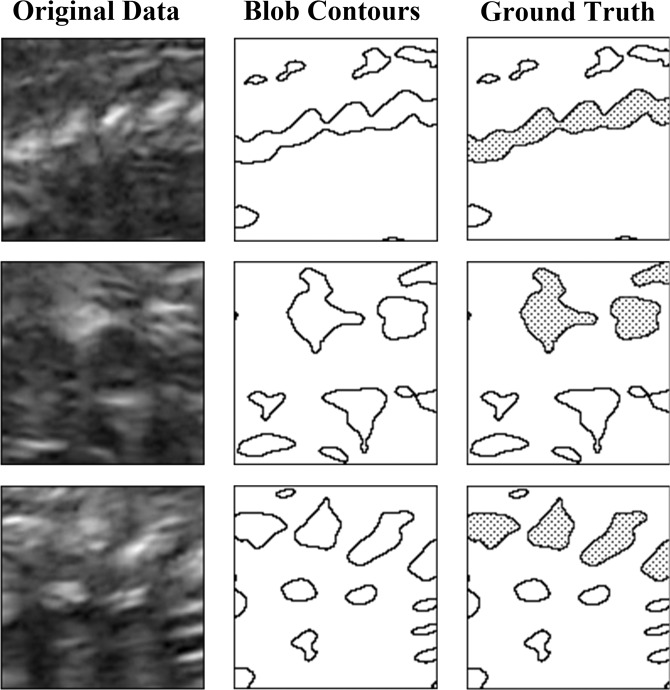
Sample regions from data set and contours of extracted binary masks. Filled blobs indicate bone regions belonging to spine.

Resulting images are binary masks (i.e. black and white images) where pixels associated with bone regions are white. To establish the ground truth, bone regions belonging to the spine are marked on all images by an expert obstetrician, which allows us to examine search space with two clusters as spine bones and non-spine bones/regions Fig. 2.

### Definition of search space

It is known that, spinal axis is located on some of these bone blobs. Each possible combination of bone regions is a candidate solution to the problem and, hence, an element of solution search space. Clearly, it is not known a priori how many spine bones are visible and distinguishable on an image. Consequently, it is also not known how many connections are required to best identify the spine. Accordingly, any algorithm aimed at locating the spine should be able to eliminate bone combinations that are too far from reality, and to identify those blobs that constitute the spine. After preprocessing, the average number of bone regions per image is found to be 70, 12–46% of which typically corresponds the spine bones. With increasing number of bone blobs in the image, the spine bones have to be identified among a larger number of candidate bones. Hence, the complexity of the spine identification problem increases with the number of bone blobs as well.

### Flocking process

Flocking behavior of the birds is one of the collective motion models that can be applied to a simulated particle system. Accordingly, one can develop an algorithm that mimics the real time swarming characteristics of bird flocks to model the formation of a virtual cluster. In such simulations, there are three principles that drives the birds, which are separation, alignment and cohesion^15^. The main objective of this study (namely, connecting bone regions belonging to the same cluster) involves connection rules that resemble the flocking principles. Bone regions belonging to spine are mostly located with a spatial pattern, i.e. along a curve, even in the most deformed cases due to SB disorder. Alignment and collective steering characteristics of the flocking dynamics are utilized to associate the spine bones with each other while eliminating non-spine regions. If neighboring bone regions that are likely to belong to the spine simultaneously can be combined into a single item from an algorithmic point of view, the search space will be significantly reduced in size. That way, the solution can be found with a smaller computational effort. The main parts of this algorithm are explained below:

#### Centering and neighboring

**Figure 3:**
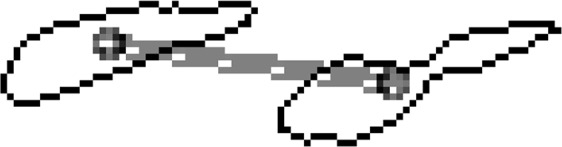
Two flocks (i.e. bone blobs) are connected through their centroids (indicated with circles) with a straight line to form a single object.

Pixels of a bone region are assumed to be the members of that flock. Accordingly, each local bone region (i.e. flock) has a centroid, whose x-y coordinate pair (*x*_*c*_, *y*_*c*_) is given by:

1a $${x}_{c}=\frac{1}{n}\mathop{\sum }\limits_{i\mathrm{=1}}^{n}\,{x}_{i}$$

1b $${y}_{c}=\frac{1}{n}\mathop{\sum }\limits_{i\mathrm{=1}}^{n}\,{y}_{i}$$

where (*x*_*i*_, *y*_*i*_) are the coordinates of the ith pixel and n is the total number of pixels in the flock. Flocks are programmed to find the best neighbor to connect. It should be noted that flocks try to find the closest neighbor. Such neighboring flocks are connected by a straight line, which can be considered a “migration path” Fig. 3).

#### Perception zone

**Figure 4:**
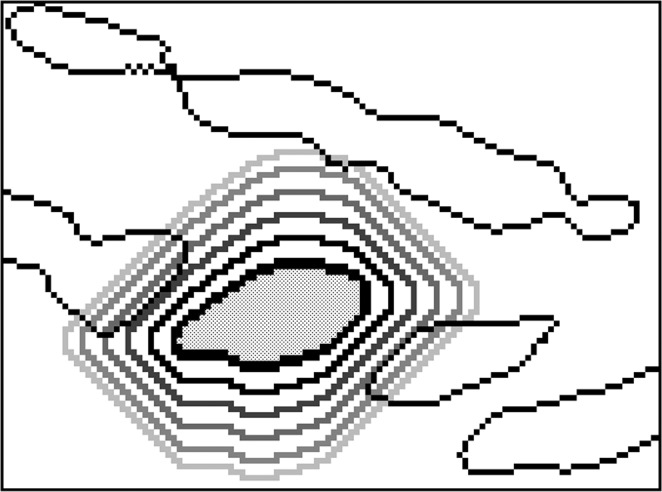
Perception zone for a sample flock. There are only three neighbors that recent flock is able to detect.

Bird flocks avoid to crowd by having a limited perception zone^16^. Hence, each bone region is assumed to have a limited perception zone and other regions are visible only if they are inside this zone. The region that each flock perceives is approximately 12 pixels from its boundary. Only those flocks that are within each other’s perception zone can be connected (Fig. 4).

#### Fatal distance

**Figure 5:**
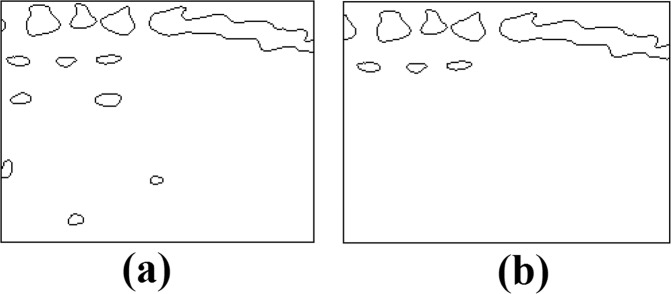
Effect of fatal distance. A sample region before (**a**) and after (**b**) eliminating isolated flocks.

It is possible that a flock could not detect any local flock-mates to connect to, if it is too isolated from all other flocks. A fatal distance is defined such that if the nearest flock is further away than this distance, the flock is terminated (Fig. 5). This effectively means that a region which is too isolated from other bone regions is not likely to be a part of the spine. The fatal distance is taken approximately as 25 pixels in this study.

#### Alignment

**Figure 6:**
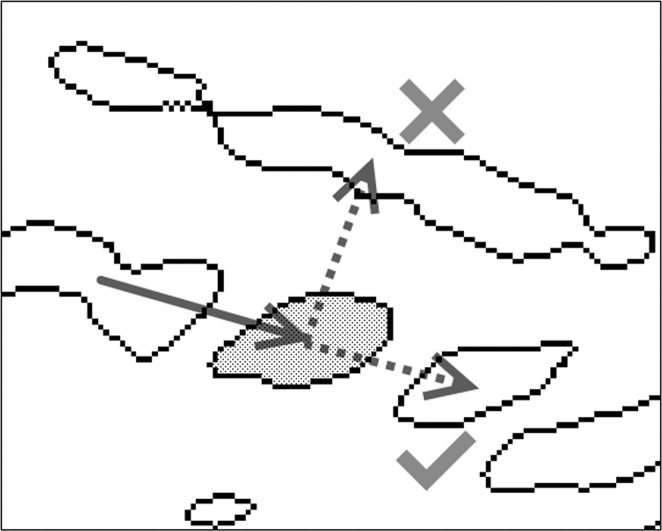
Choice of best neighbor according to orientation. The best flock to connect is looked for the darker colored flock. The previous connection to a flock (marked with a cross) is terminated in favor of a flock (marked with a check) which is likelier to belong to the spine.

Proposed algorithm searches for the best neighbor to connect for each flock. It should be noted that orientation of the connections that two or more flocks make with each other will affect the choice of the next neighbor to be added to this flock group (alignment principle)^17^. This allows local flocks that align along a smooth curve to be grouped together (since aligned bone regions are likelier to belong to the spine). Fundamental steps for determining the flocks to be connected is given in Algorithm 1 and visualized in Fig. 6.

**Algorithm 1:**
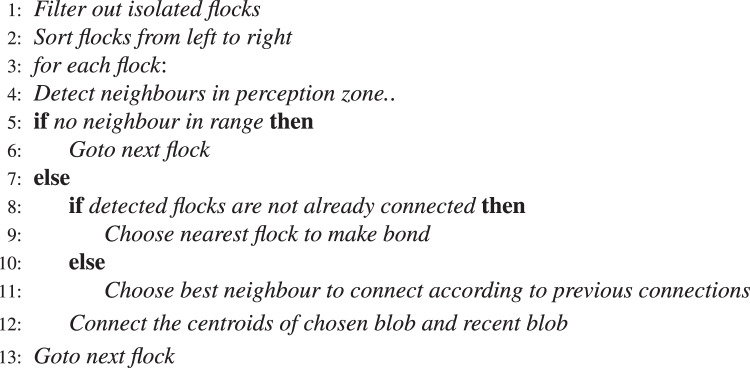
Determination of neighbours to connect.

### Genetic algorithm for the evaluation of flocking based search space reduction

The function of the utilized GA is merely to evaluate the performance of the search space reduction by comparing the convergence of reduced and not-reduced search spaces. Hence, a straightforward GA with an idealized, ground-truth-based fitness is utilized to prevent any complications associated with the GA and emphasize the performance of the search space reduction procedure.

For the same sample, the GA is run with and without search space reduction. The classification process in both cases is based on the evolution of an initial population and it is accomplished via common genetic operators (REF). For completeness, some important concepts of GA are explained in connection with the current problem below:

**Individuals:** Each candidate solution to the problem of spine identification is an individual. Individuals consist of genes.

**Genes:** Each flock region is represented by a gene in the candidate solution (i.e. individual). Accordingly, for a given problem (i.e. an ultrasound sample), each individual has a fixed number of genes (representing the number of visible spine bone regions in the ultrasound sample)^18^. A gene is a binary variable, which equals one in case the related flock belongs to spine and zero otherwise.

**Population:** A population is the collection of a predefined number of candidate solutions^19^. In this work, the population consists of 20 individuals.

**Generation:** Population renewed with evolutionary operators forms the next generation in the GA^20^. Maximum number of generations is a predefined parameter. Reaching to maximum number of generations is accepted as a stop criteria in the algorithm.

### Evolution mechanism

**Figure 7:**
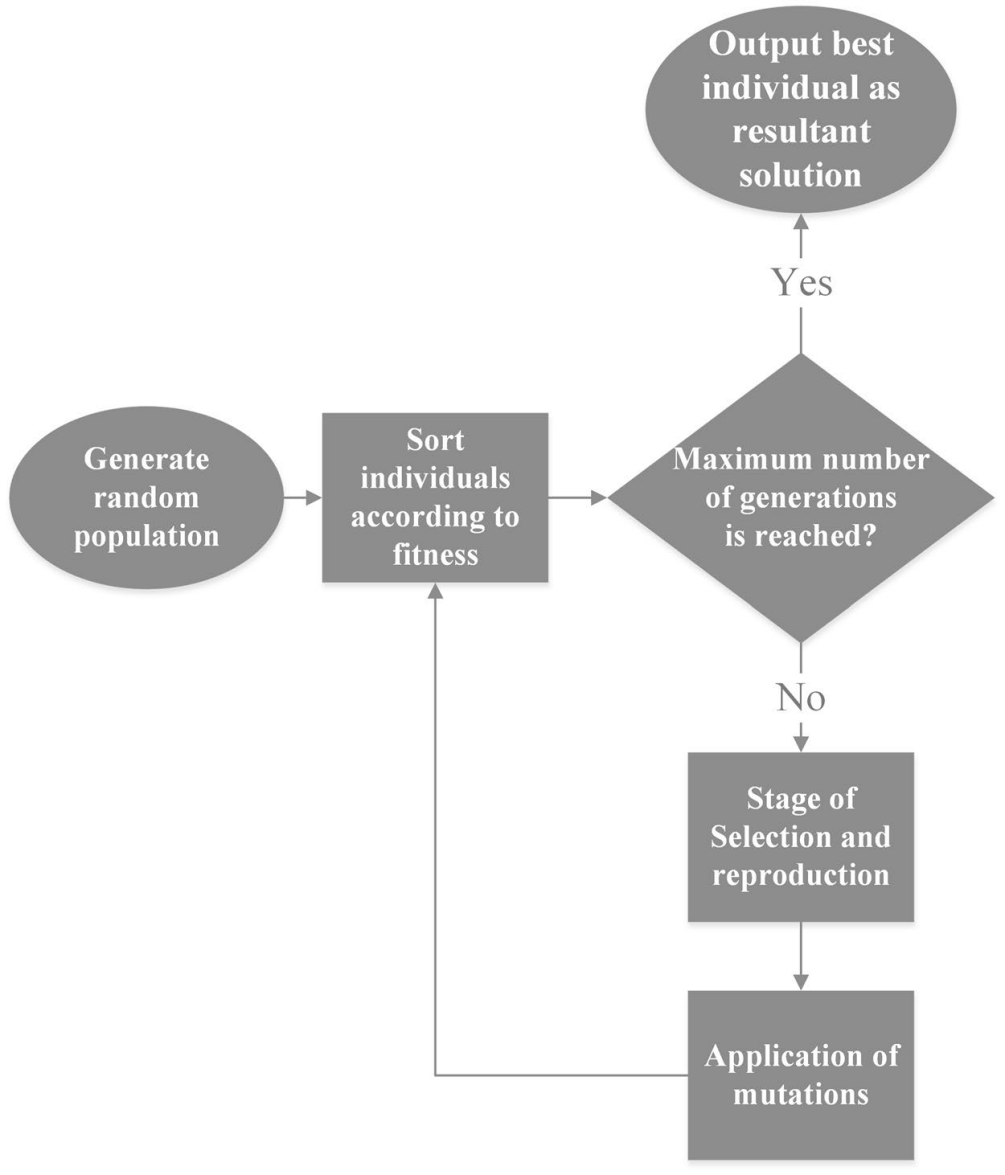
Flowchart of the implemented conventional genetic algorithm. At every generation, the best individuals are found and passed to the next generation. Unless the maximum generation number is reached, the best individuals are passed to the crossing-over and mutation operations to form the next generation individuals.

Bone groups of the reduced search space are randomly assigned to individuals as spine or non-spine to form the initial population. The evolution of a population is then realized by applying two genetic operators to the population at every iteration (representing a generation). The best two-individuals go automatically to the next generation. Using the remaining 18 individuals, 18 new individuals are created through crossing-over and passed also to the next generation. (While essentially a random process, better individuals are given a higher probability of undergoing crossing-over.) A randomly chosen half of the 20 individuals then undergo a mutation where one random gene in each individual is flipped.(In other words, a spine is turned into non-spine or vice versa.) The crossing-over operator allows the best solutions to pass their properties to the subsequent iterations and the mutation operator diversifies the population to increase the chance of finding fitter solutions (Fig. 7). The fitness criteria is based on the known ground truth and detailed in the next subsection.

### Fitness criteria

The fitness value is an indicator for the closeness of the individual to the actual solution^21^. In many problems where GAs are utilized, the best solution (or whether there exist a best solution or not) is not known. In this work, however, the best solution is known and each individual in each generation is compared with the best solution and assigned a fitness value accordingly. The fitness of an individual is measured through the number of correctly identified spine bones. The F-Score with respect to the ground truth is defined as:

2a $$F-score=2\ast \frac{Precision\ast Recall}{(Precision+Recall)}$$

where,

2b $$Precision=\frac{Tp}{(Tp+Fp)}$$

2c $$Recall=\frac{Tp}{(Tp+Fn)}$$

Here, *Tp*, *Fp* and *Fn* indicate the number of true positives (correctly identified spine), number of false positives (non-spine identified as spine) and number of false negatives (spine identified as non-spine, respectively^22^.

### Possible error sources and evaluation

In the search space reduction methodology, there are two possible failure scenarios that can affect the performance of the search space reduction procedure. One of them is the possibility of a false connection. Connections of bones belonging to the correct cluster (either spine or non-spine) should be considered a true connection. For example, two or more spine regions connected or equivalently two or more non-spine regions connected should be considered optimal. The other possible scenario is the false elimination (deletion) of a spine bone. If any spine blob is erased falsely due to its distance to a nearest neighbor, success of the utilized classifier would be compromised. Hence, the performance of the search space reduction algorithm is first evaluated by observing the number of false connections, false eliminations and the reduction in the search space. The reduction is measured using the number of bone regions before and after the reduction process. For example, if the number of bone regions is 100 and 70 before and after reduction, respectively, this corresponds to a 30% reduction in search space size.

Once the search space is reduced, the spine axis is estimated by the GA algorithm. As explained above, the performance of the GA in identifying the spine is achieved by calculating the F-score. Clearly, in case of false connection and false elimination, the GA can never reach the perfect solution (i.e. ground truth). Yet, it may happen in some samples that, despite a seemingly large number of false connections (and/or eliminations) in the sample, the estimated spine axis looks still close to the actual one. In this case, the number of falsely connected bone regions alone may be misleading with respect to the success of the search space reduction scheme. This occurs typically when falsely connected bones are relatively small in size and/or located close to the actual spine axis. Consequently, we have decided to define an error measure that reflects the accuracy of the entire spine axis as well. For evaluation purposes, a fifth order polynomial is fitted to the centroids of the spine regions on each image. We refer to this polynomial as the ground truth polynomial, since it is the continuous representation of the actual spine. A fifth order polynomial is adequate to capture the shape of the actual spine. An interpolation polynomial too high of an order through a relatively small number of points may be oscillatory and loose the essence of interpolation, a phenomenon referred to as Runge phenomenon in numerical analysis^23^. Next, a fifth order polynomial is fitted to the group centroids of the spine identified by the algorithm and a comparison is made with the ground truth polynomial via root mean squared error (RMSE) defined by:

3 $$RMSE=\sqrt{\frac{\mathop{\sum }\limits_{i\mathrm{=1}}^{n}{({y}_{i}-f({x}_{i}))}^{2}}{n}}$$

where (*x*_*i*_, *y*_*i*_) indicate the coordinates of the points of the estimated curve. *f*(*x*_*i*_) is the corresponding y value of the ground truth polynomial. The error is evaluated in an integral sense and thus n is the number horizontal pixels in the x-range of the spine. Simultaneously, three other expert obstetricians, who has not seen the filtered images, draw the spine axis by hand on each raw ultrasound image. This is achieved via a digitizer tablet with pen whose movement resolution is 2540 lpi. The deviation of these curves from the ground truth polynomial is also measured by calculating their RMSE value.

### Ethics approval and consent to participate

The authors declare that this study does not contain any personal information that could lead to the identification of the patients and Informed consent was obtained from all participants. The work described has been carried out in accordance with the ethical approval of the Çukurova University Faculty of Medicine, Non-Interventional Clinical Research Ethics Committee.

### Consent for publication

All authors read and approved the manuscript.

## Results

### Results of the search space reduction algorithm

**Figure 8:**
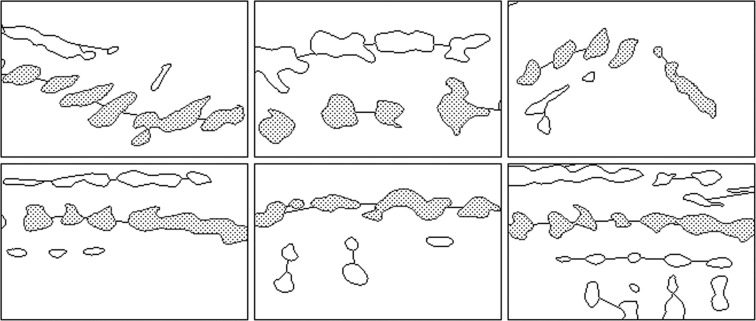
Output of flocking shown on different samples. Filled regions are bones on the spine axis where migration paths are shown with lines.

The search space reduction algorithm along with the GA is run for the fourteen fetal ultrasound images. Typical output of search space reduction process is presented for six different sample regions in Fig. 8. Spine bones are indicated by filled blobs while non-spine regions are shown as empty contours in the figure.

Experimental results indicate that the reduction in search space size varies significantly among samples. The percent reduction can be as low as 26% or as high as 68% Table 1.

**Table 1:** Percent reduction of search space given for each sample with false connections and false removal rates.

	% Reduction in Search Space	Number of Connections	False Connection Rate	False Removal Rate
Sample 1	29	32	0	0
Sample 2	32	26	0	0
Sample 3	43	44	5	0
Sample 4	26	43	16	0
Sample 5	65	14	0	0
Sample 6	33	13	0	0
Sample 7	61	26	0	0
Sample 8	35	11	0	0
Sample 9	50	23	17	0
Sample 10	34	39	0	0
Sample 11	35	21	5	0
Sample 12	33	25	0	0
Sample 13	47	27	11	0
Sample 14	68	16	0	0

The sample images have different complexities and this variation is found to be normal in that sense. False elimination (deletion) of a spine bone on any of the samples is not observed. However, false connections (i.e. a non-spine bone connected with a spine bone) are observed on five of the fourteen samples.

### Results of the spine identification with the genetic algorithm

The GA algorithm is tried with fourteen different image samples for 100 generations on both optimized (reduced number of bone regions) and non-optimized (original ultrasound sample) version of each image sample. Due to intrinsic randomness present in GA, each run is repeated ten times and mean values of F-scores and their standard deviation are calculated for comparison (Tables 2 and 3).

**Table 2:** Mean F-Scores are presented for several generations.

	Non-optimized				Optimized			
	Gen:1	Gen:20	Gen: 50	Gen:100	Gen:1	Gen:20	Gen: 50	Gen:100
Sample 1	0.711	0.804	0.866	0.930	0.859	0.965	0.996	1.000
Sample 2	0.636	0.763	0.853	0.919	0.752	0.956	0.995	1.000
Sample 3	0.695	0.784	0.841	0.912	0.777	0.889	0.939	0.972
Sample 4	0.667	0.788	0.868	0.933	0.771	0.896	0.922	0.932
Sample 5	0.712	0.820	0.882	0.933	0.805	0.893	0.935	0.979
Sample 6	0.738	0.865	0.931	0.976	0.905	0.992	1.000	1.000
Sample 7	0.687	0.784	0.847	0.909	0.759	0.859	0.933	0.974
Sample 8	0.710	0.858	0.933	0.983	0.876	0.971	1.000	1.000
Sample 9	0.682	0.801	0.886	0.940	0.795	0.888	0.925	0.944
Sample 10	0.720	0.818	0.880	0.942	0.820	0.940	0.988	0.996
Sample 11	0.750	0.880	0.940	0.988	0.860	0.973	0.978	0.980
Sample 12	0.732	0.857	0.924	0.976	0.836	0.984	1.000	1.000
Sample 13	0.702	0.791	0.851	0.924	0.799	0.888	0.933	0.963
Sample 14	0.682	0.804	0.858	0.929	0.760	0.850	0.928	0.984

**Table 3:** Standard deviations of mean F-Scores are presented for several generations.

	Non-optimized				Optimized			
	Gen:1	Gen:20	Gen: 50	Gen:100	Gen:1	Gen:20	Gen: 50	Gen:100
Sample 1	0.042	0.030	0.032	0.023	0.024	0.023	0.007	0.000
Sample 2	0.049	0.037	0.034	0.036	0.041	0.035	0.011	0.000
Sample 3	0.043	0.029	0.037	0.028	0.044	0.031	0.023	0.016
Sample 4	0.034	0.030	0.025	0.022	0.041	0.024	0.017	0.004
Sample 5	0.054	0.042	0.031	0.022	0.036	0.025	0.012	0.011
Sample 6	0.042	0.031	0.033	0.013	0.020	0.009	0.000	0.000
Sample 7	0.021	0.016	0.015	0.018	0.042	0.032	0.026	0.012
Sample 8	0.066	0.054	0.044	0.012	0.045	0.022	0.000	0.000
Sample 9	0.031	0.022	0.036	0.035	0.029	0.021	0.016	0.010
Sample 10	0.021	0.042	0.030	0.021	0.050	0.029	0.012	0.009
Sample 11	0.037	0.048	0.035	0.019	0.043	0.013	0.006	0.000
Sample 12	0.032	0.036	0.022	0.015	0.071	0.028	0.000	0.000
Sample 13	0.024	0.024	0.025	0.016	0.030	0.022	0.022	0.015
Sample 14	0.061	0.046	0.031	0.025	0.034	0.038	0.020	0.011

**Figure 9:**
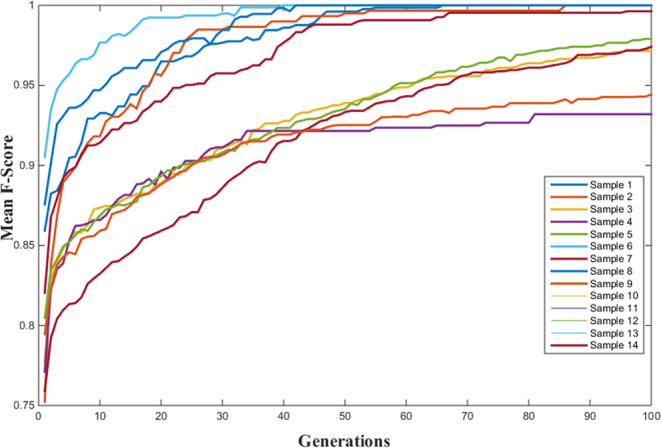
Classification performance of genetic setting is given in different colors for each sample. Mean of ten repetitions is given for each generation.

In most of the image samples, the optimized version consistently results to a better and faster convergence to the ground truth solution. The iteration converges to a value of 1 if spinal bone regions are connected correctly, which is the case in most samples (Table 2). The standard deviation is generally low and decreases further with increasing generation number (Table 3). An F-score value smaller than 1 implies that either convergence has not occurred yet or a non-spine region is connected to a bone group. Since the visual complexity of each ultrasound sample image is generally different, convergence rate may differ significantly from one sample to the other. The convergence behavior of different ultrasound samples are comparatively displayed in Fig. 9.

### Results regarding the continuous spine axis

**Figure 10:**
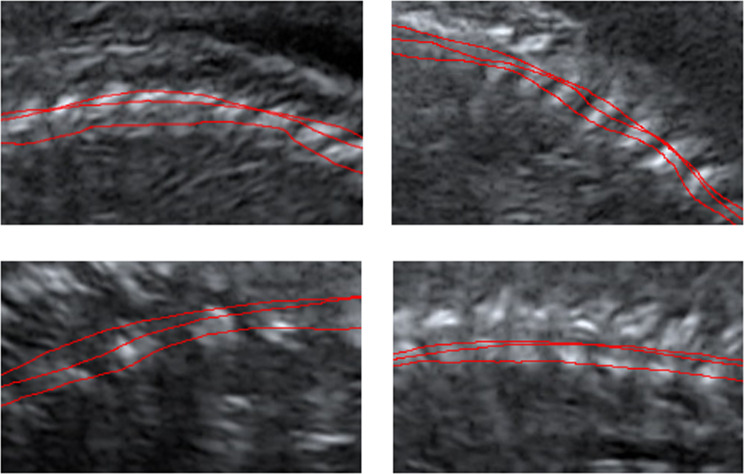
Curves hand drawn by experts shown on sample regions.

The curves drawn by the expert by hand on the spinal axis possess some error compared with the fifth order ground truth polynomial, since the experts do no try pass through the bone region centroids (Fig. 10).

The RMSE range of expert-curves is between 5 and 50 pixels. The interpolation polynomial fit to the estimated spine axis usually has a comparable RMSE to the expert curves if not better (Table 4).

**Table 4:** Integrated RMSE values of hand drawn curves and estimated curve with respect to the ground truth polynomial.

	Number of False Connected Blobs	Exp. 1	Exp. 2	Exp. 3	Estimated Curve after Optimization
Sample 1	0	12.8	31.9	7.2	3.9
Sample 2	0	21.6	9.6	5.6	1.8
Sample 3	2	11.6	15.5	18.3	8.5
Sample 4	10	9.0	14.4	11.6	7.0
Sample 5	0	18.6	49.5	46.6	0.8
Sample 6	0	7.2	11.8	18.8	1.0
Sample 7	0	12.0	16.2	15.2	4.2
Sample 8	0	9.9	32.4	14.2	0.3
Sample 9	6	7.9	6.3	15.8	8.1
Sample 10	0	7.7	12.3	17.1	0.3
Sample 11	1	13.6	15.9	12.7	0.7
Sample 12	0	3.3	3.9	3.2	0.5
Sample 13	3	20.4	10.1	9.7	12.1
Sample 14	0	6.5	8.1	9.7	3.6

This observation is valid even for those samples where there exist falsely connected blobs, indicating that existence of false connections does not necessarily result to a poorly estimated spine axis.

## Discussion and Conclusions

In this study, we have proposed a novel nature inspired methodology for reducing the search space in the spine identification problem associated with the ultrasound images of SB cases. The proposed search algorithm has been seen to combine bone regions into groups effectively to reduce the total number of bone blobs forming the search space. The reduction rate is very much dependent on the nature of the sample image and has varied between 26% and 68% for the samples used (Table 1). Even with a moderate decrease in the number of bone regions, the possible number of combinations that would make a spine can decrease typically several orders of magnitude, which is a more significant simplification than the percentage value reflects. Hence, this reduction in search space increases the effectiveness of the GA drastically.

In this research, the GA is merely used to demonstrate the effectiveness of the search space reduction. To prevent any performance loss associated with the GA, it based on a known ground truth. In such a case, a GA is guaranteed to capture the ground truth eventually. In an actual spine identification problem, an appropriate fitness function needs to be proposed, such that the GA can cluster the bone groups, “pre-clustered” by the search space reduction algorithm, into spine and non-spine bones. We have developed and evaluated such a fitness function in a follow-up study, which will be submitted as another manuscript in the coming days. The search space reduction algorithm has connected blobs without any interclass (i.e. spine with non-spine) connections in nine out of fourteen samples. False elimination (i.e. removal of isolated spine blobs) has not been observed in any of the samples. Even if there are false connections in one sample image, the interpolation polynomial that is fit on the identified spine is typically close to the ground truth polynomial and to the expert-curves drawn on the raw ultrasound images. This observation is related with the fact that falsely connected bone regions are typically small and do not shift the centroid location of the bone group very much. It should be noted that the ground truth polynomial passes through the centroids of the individual spine bones, whereas the interpolation polynomial passes through the centroids of the grouped bones. Even if all spine bones are correctly identified in the search space reduction, the interpolation polynomial will pass through the group centroids and deviate slightly from the ground truth polynomial. This is why we have a small but non-zero RMSE for samples that have no false connections (for example, Samples 1, 2, 5 and 6 on Table 4). For all practical purposes, these samples can be considered error-free.

In this study, we have worked on SB cases due to its clinical significance but the algorithm can work on healthy spine samples as well. We have experimented on healthy samples and observed that the performance of the algorithm has been comparable to the SB cases.

While this study does not intend to propose a computer aided diagnosis (CAD) system for SB, the proposed methodology, together with an appropriate fitness function, can constitute the first natural stage of such a CAD system.

## Data Availability

Ultrasound samples used in this study are available from the corresponding author on reasonable request.
